# An integrated multi-tissue approach for endometriosis candidate biomarkers: a systematic review

**DOI:** 10.1186/s12958-023-01181-8

**Published:** 2024-02-10

**Authors:** Axelle Brulport, Mathilde Bourdon, Daniel Vaiman, Christian Drouet, Khaled Pocate-Cheriet, Kheira Bouzid, Louis Marcellin, Pietro Santulli, Carole Abo, Maxime Jeljeli, Sandrine Chouzenoux, Charles Chapron, Frédéric Batteux, Camille Berthelot, Ludivine Doridot

**Affiliations:** 1 Institut Pasteur, Université Paris Cité, CNRS UMR 3525, INSERM UA12, Comparative Functional Genomics Group, Paris, 75015 France; 2 Université Paris Cité, Institut Cochin, INSERM, CNRS, F-75014 Paris, France; 3 Département de Gynécologie, Obstétrique et Médecine de la Reproduction, AP-HP, Centre Hospitalier Universitaire (CHU) Cochin, F-75014 Paris, France; 4 Université de Paris, Faculté de Santé, Faculté de Médecine Paris Centre, Paris, France, Service de Biologie de la Reproduction - CECOS, AP-HP, Centre Hospitalier Universitaire (CHU) Cochin, Paris, 75014 France; 5 Service d’Immunologie Biologique, AP-HP, Centre Hospitalier Universitaire (CHU) Cochin, Paris, F-75014 France

**Keywords:** Endometriosis, Candidate biomarkers, Biological compartments, Endometriosis phenotypes

## Abstract

Biomarker identification could help in deciphering endometriosis pathophysiology in addition to their use in the development of non invasive diagnostic and prognostic approaches, that are essential to greatly improve patient care. Despite extensive efforts, no single potential biomarker or combination has been clinically validated for endometriosis.

Many studies have investigated endometriosis-associated biological markers in specific tissues, but an integrative approach across tissues is lacking. The aim of this review is to propose a comprehensive overview of identified biomarkers based on tissue or biological compartment, while taking into account endometriosis phenotypes (superficial, ovarian or deep, or rASRM stages), menstrual cycle phases, treatments and symptoms.

We searched PubMed and Embase databases for articles matching the following criteria: 'endometriosis' present in the title and the associated term 'biomarkers' found as Medical Subject Headings (MeSH) terms or in all fields. We restricted to publications in English and on human populations. Relevant articles published between 01 January 2005 (when endometriosis phenotypes start to be described in papers) and 01 September 2022 were critically analysed and discussed.

Four hundred forty seven articles on endometriosis biomarkers that included a control group without endometriosis and provided specific information on endometriosis phenotypes are included in this review. Presence of information or adjustment controlling for menstrual cycle phase, symptoms and treatments is highlighted, and the results are further summarized by biological compartment. The 9 biological compartments studied for endometriosis biomarker research are in order of frequency: peripheral blood, eutopic endometrium, peritoneal fluid, ovaries, urine, menstrual blood, saliva, feces and cervical mucus. Adjustments of results on disease phenotypes, cycle phases, treatments and symptoms are present in 70%, 29%, 3% and 6% of selected articles, respectively. A total of 1107 biomarkers were identified in these biological compartments. Of these, 74 were found in several biological compartments by at least two independent research teams and only 4 (TNF-a, MMP-9, TIMP-1 and miR-451) are detected in at least 3 tissues with cohorts of 30 women or more.

Integrative analysis is a crucial step to highlight potential pitfalls behind the lack of success in the search for clinically relevant endometriosis biomarkers, and to illuminate the physiopathology of this disease.

**Graphical Abstract:**

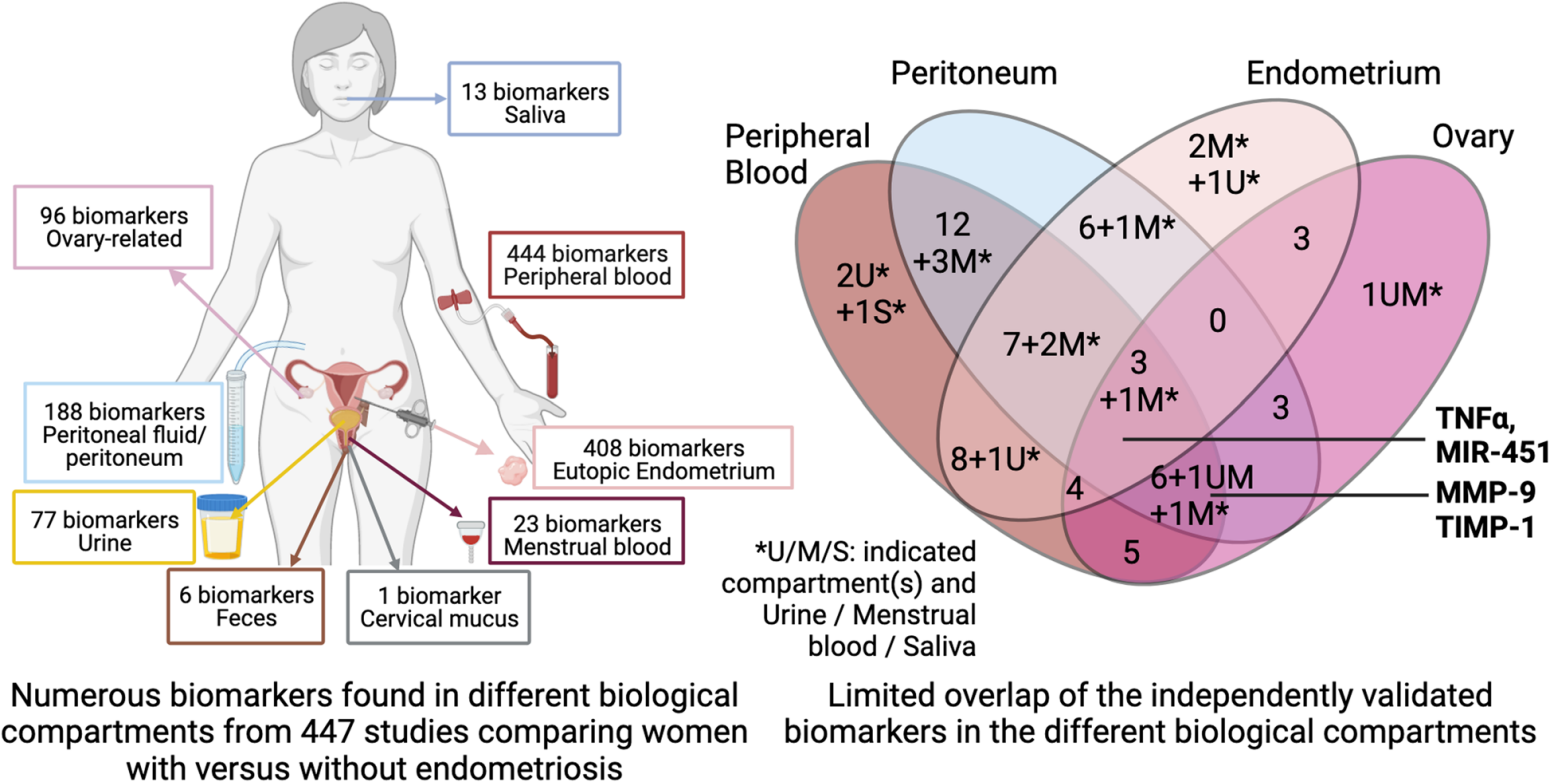

**Supplementary Information:**

The online version contains supplementary material available at 10.1186/s12958-023-01181-8.

## Introduction

Endometriosis is a chronic gynecological condition affecting 6%-10% of women of reproductive age [1]. Histologically, endometriosis corresponds to the dissemination of endometrial-like tissue, or lesions, outside the uterus. The reference method to diagnose endometriosis is surgery, through lesion visualisation and anatomical pathology evaluation. Endometriosis staging is currently based on surgeons' observations. The most widely used scoring is from the American Society for Reproductive Medicine (ASRM) that ranges endometriosis from stage I “minimal” to stage IV “severe”, based on lesion localisation, size, appearance and presence of adhesions [2]. Currently, patient management practices promote non-invasive diagnostic methods such as transvaginal ultrasonography and magnetic resonance imaging (MRI) [3]. Not all staging features are accessible with non-invasive diagnostic approaches, which makes the ASRM classification difficult to use in this context. An alternative classification defines three phenotypes: superficial peritoneal lesions (located less than 5 mm below the peritoneum), ovarian endometriomas, and deep infiltrating endometriosis (located more than 5 mm below the peritoneum). The last two can usually be detected by imaging.

Clinically, endometriosis is associated with a wide range of symptoms and consequences: pelvic pain, severe pain during periods (dysmenorrhea), painful sex, painful urination (dysuria) and/or defecation (dyschesia), alternance of diarrhea/constipation, heavy menstrual bleeding, mood disorders, chronic fatigue and infertility [4]. Endometriosis is increasingly considered as a systemic disease rather than a pelvic pathology [5].

Because of its complex pathophysiology, symptoms heterogeneity, and diagnostic requirements, diagnosis delay for endometriosis ranges from 4 to 11 years [5]. Biomarker candidate research is therefore a key avenue to improve diagnosis. Despite extensive efforts, no single or combination of biomarkers has reached clinical validation for endometriosis, as extensively reviewed in Cochrane’s reviews in 2016 [6–9]. While dozens of potential biomarkers have been investigated across varied biological compartments, few have been validated in independent studies, making their relevance unclear. To address this, we propose an original integrative review of the endometriosis biomarker literature across biological compartments. Our hypothesis is that endometriosis biomarkers recurrently identified across multiple tissues may be particularly relevant and play a more direct role in disease physiopathology, and an integrative multi-tissue approach could highlight and prioritize these candidates, which may eventually lead to enhanced patient care. For all considered studies, we highlight if endometriosis subtypes, menstrual cycle phases, treatments and symptoms were accounted for. We particularly focus on biomarkers reproducibly detected by at least two independent research teams, and found in different biological compartments.

## Materials and methods

### Literature search

Pubmed and Embase (excluding Medline articles) were searched for English-language articles as follows: Endometriosis in the title AND ‘biomarkers’ as Medical Subject Heading (MeSH) or All Fields terms. The strategy was designed in association with the referral Inter-University Library of Medicine of Université Paris Cité, France. All articles involving human subjects and published between 2005/01/01 and 2022/09/01 were selected for screening. The databases were last consulted on 30 September 2022. The review was conducted in accordance with The PRISMA 2020 statement for systematic review and not registered [10]. PRISMA 2020 Checklist is included in Additional Material.

### Eligibility criteria and study selection

**Fig. 1:**
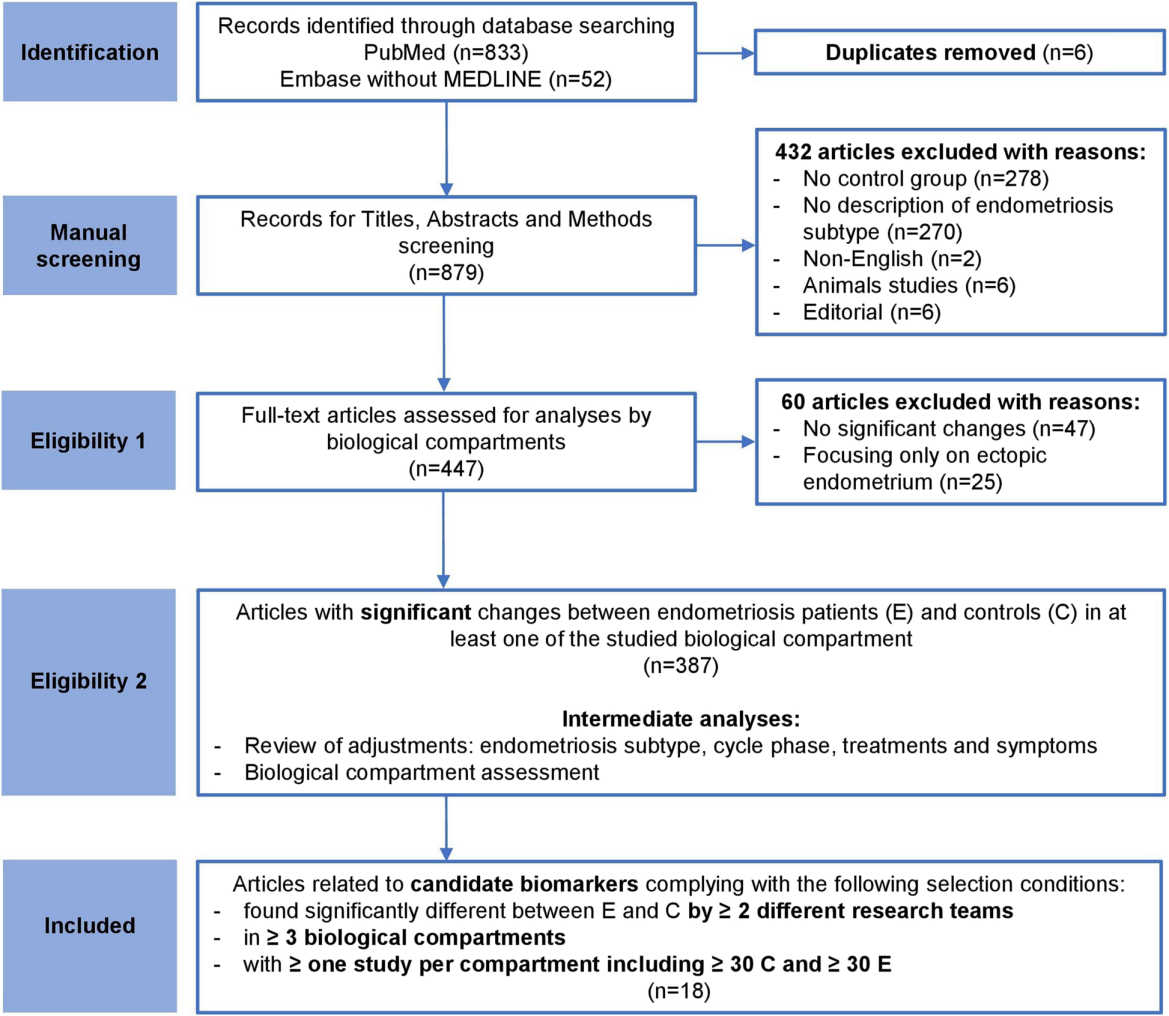
PRISMA flowchart for the systematic review. Flowchart highlighting the different steps in the selection of articles included in the review, giving details of the inclusion and exclusion criteria and the analyses carried out

The study selection strategy is summarized as a flowchart in Fig. 1. Both clinical and basic research studies were considered. A total of 879 original publications were manually screened. At each step of the selection process, titles/abstracts/methods or full-text were screened by 2 independent reviewers (A.B. and one of the other co-authors) regarding eligibility criteria. Discrepancies concerning studies inclusion were resolved by A.B. and L.D. All review articles, editorial, animal subject studies and publications not written in English were excluded. Articles with the two following mandatory inclusion criteria were selected for further evaluation: i) presence of a control group without endometriosis and without malignant diseases, and ii) available information on endometriosis phenotype(s). For the remaining 447 articles, the full text was screened to extract the biomarkers studied, the significance and the direction of the variation observed between control and endometriosis groups and the biological compartment in which these changes were found. Only biomarkers significantly deregulated in endometriosis were considered. Biomarkers deregulated in ectopic endometrium only, and articles focusing exclusively on ectopic endometrium were excluded from this review because ectopic endometrium has no equivalent tissue in the control group. At this step, we obtained a list of 387 articles with 1107 significantly deregulated biomarkers in endometriosis and affected biological compartment(s). This list from the remaining 387 publications, validated independently by 2 reviewers, was reviewed a third time to extract information and/or adjustments for disease phenotypes, menstrual cycle phases, treatments and symptoms, which represent parameters of major importance in endometriosis. A list of candidate biomarkers per compartment was also created. Identified biomarkers and their instances in each compartment were tallied using a custom Perl script identifying unique character strings (case insensitive). To focus on sustained multi-tissue evidence, we selected articles related to the biomarkers identified by at least 2 different research teams (regardless of biological compartment) and across at least 3 biological compartments. For each candidate biomarker, at least one study per compartment including 30 or more controls and 30 or more patients with endometriosis was mandatory. All extracted data were analysed descriptively.

### Data extraction and analysis

The data related to our final set of candidate biomarkers of interest in this review were extracted from 18 articles (see flowchart in Fig. 1). For each article, the following data were extracted by 2 independent reviewers: the quality of the control groups and the homogeneity of the confounding factors between the groups, the level of expression (mRNA, protein) and the direction and amplitude of variation of the candidate biomarker, the adjustments of the results according to the subtypes of endometriosis, the phase of the menstrual cycle, the treatments and the symptoms and the ROC curve analysis to highlight diagnostic accuracy if available.

### Assessment of risk of bias

Due to the great disparity in study designs (clinical and basic research) available for each candidate biomarkers of interest, we did not use ROC curve analysis as a selection criterion to address robustness, but instead included the presence of at least one study per biological compartment including at least 30 individuals per group as a mandatory criterion. For the creation of lists of candidate biomarkers by biological compartments, to avoid computing different aliases of the same gene/protein as different biomarkers, all identified candidate biomarkers were manually curated to unify writing styles and conventions before processing. Aliases were identified through the HUGO Gene Nomenclature Committee (HGNC) Multi symbol checker tool (https://www.genenames.org/tools/multi-symbol-checker/), replacing 76 aliases by their official gene symbols.

## Results

### Extensive but heterogeneous studies have explored potential endometriosis biomarkers

We systematically searched the PubMed and Embase databases for research articles on endometriosis biomarkers published between January 2005 and September 2022. Of the 879 publications retrieved after exclusion of duplicates, 278 focused on comparisons i) among endometriosis patients or between tissues in endometriosis patients or ii) between endometriosis and cancer patients (Fig. 1). We excluded these articles since their usability for endometriosis diagnosis is limited. 270 articles did not include information on the patient endometriosis phenotypes according to either the rARSM classification or lesions localization (Fig. 1). These articles were also excluded, as we chose to study how each candidate biomarker was potentially relevant to specific subtypes of patients. In total, we retained 447 publications for further analysis (Additional Table 1), of which 387 identified at least one biomarker with significantly modified levels in endometriosis patients compared to controls (Fig. 1).

### Cycle phase, treatments and symptoms are rarely accounted or adjusted for

**Fig. 2:**
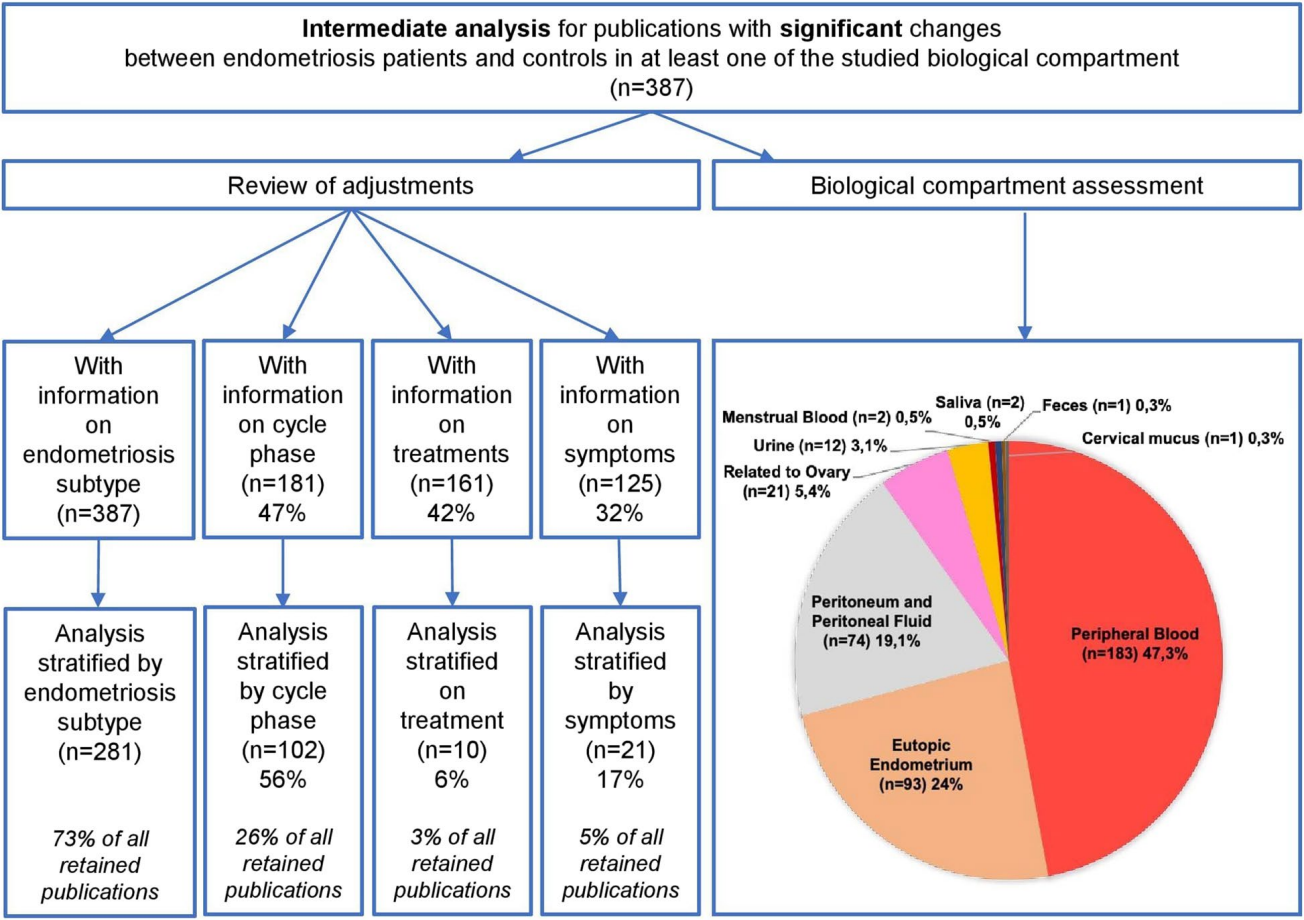
Intermediate analyses carried out on 387 articles. Analyses performed i) on the adjustments of the results according to the subtype of endometriosis, the menstrual cycle phases, the treatments and the symptoms and ii) on the different biological compartments studied

Information on endometriosis phenotypes was a mandatory inclusion criterion in this study, and 73% of selected publications adjusted the results accordingly, either intentionally or indirectly by including only a particular phenotype (Fig. 2). Biomarker levels can vary with menstrual cycle phases [11], but only 47% of selected publications provided information about cycle phase. Just over half took this parameter into account when analysing the results (Fig. 2), and mainly because surgical teams operated on patients either in the follicular phase or in the luteal phase. Regarding treatments, 42% of articles provided some information (Fig. 2). Non-use of hormonal treatments in the 3 to 6 months prior to inclusion is often specified (and sometimes non-use of anti-inflammatory drugs in the days before inclusion), explaining why only 3% of the analysed publications adjust results for hormonal or symptomatic treatments (anti-inflammatory, painkillers, etc.; Fig. 2). Although endometriosis symptoms are very diverse, this aspect is the least documented, with only 32% of analysed articles taking symptoms into account (Fig. 2). Of these, 17% reported subgroup analyses depending on symptoms (Fig. 2). Infertility, a consequence of endometriosis often regarded as a symptom, was the most commonly considered.

### One thousand one hundred seven biomarkers identified across nine unequally studied biological compartments

Among 447 retained publications, 387 identified a significant biomarker in at least one biological compartment (Fig. 1 and 2). The majority studied peripheral blood (183 articles, 47,3%) (Fig. 2, Additional Table 2). Other studied compartments were eutopic endometrium (93 articles, 24%), peritoneum and peritoneal fluid (74 articles, 19,1%), ovary (mainly follicular fluid or cumulus cells; 21 articles, 5,4%), urine (12 articles, 3,1%), menstrual blood (2 articles, 0,5%), saliva (2 articles, 0,5%), feces (1 article, 0,3%) and cervical mucus (1 article, 0,3%) (Fig. 2). 444 biomarkers were identified in peripheral blood, 408 in eutopic endometrium, 188 in peritoneum and peritoneal fluid, 96 in compartments related to ovary, 77 in urine, 23 in menstrual blood, 13 in saliva, 6 in feces and 1 in cervical mucus (Additional Table 2). In total, we listed 1107 candidate biomarkers of endometriosis, several of which were identified in different compartments. Interestingly, only a minority were reproducibly found in independent articles within the same compartment (Additional Table 2), questioning the standardisation and reproducibility of endometriosis candidate biomarker studies. Moreover, only 74 were found in several biological compartments by at least two independent research teams (Table 1). These 74 biomarkers significantly modified in endometriosis were classified into molecular subtypes (Table 1) and used for the following selections. The names of the 74 candidate biomarkers identified, their direction of variation in the different biological compartments studied and the cohort sizes of the selected articles are detailed in Table 1.

**Table 1:** Biomarkers found in different biological compartments by independent teams

Molecular subtypes	Biomarkers found in several tissues	Biological compartments							References by compartment: B for Blood, P for Peritoneum/Peritoneal fluid, E for Endometrium, O for ovary, U for Urine, M for menstrual blood, and Sa for Saliva
		B	P	E	O	U	M	Sa	*n* = X&Y with X the number of samples from women without endometriosis, Y the number of samples from women with endometriosis
									Up/Down (in endometriosis patients vs controls) is indicated when studies are inconsistent
Immunity related markers / Cytokines	CXCL8 (IL8)	↑	↑	↑	↓*		↑*		B: [12] ***n* = 93&201**; [13] *n* = 25&19; [14] *n* = 12&75 P: [15] ***n* = 30&48**; [16] *n* = 16&67; [17] *n* = 20&57; [18] *n* = 27&36; [19] ***n* = 40&58**; [20] ***n* = 34&124**; [21] ***n *= 38&56**; [22] ***n* = 45&126**; [23] ***n* = 35&45** E: [24] *n* = 11&24; [25] *n* = 8&15; [26] *n* = 5&5 O: [27] *n* = 9&9 (cumulus cells) – M: [28] *n* = 3&3
	**TNFa**	↑↓	↑	↑	↑				B: Up [13] *n* = 25&19; **Up**[29]***n* = 103&190**, **Down**[12]***n* = 93&201**; **Down **[30]***n* = 121&232** P: [31] *n *= 22&30; [32] *n* = 17&33; [17] *n* = 20&57; [23] ***n* = 35&45**; [33] ***n* = 59&73** E: [24] *n* = 11&24 – O: [34] ***n* = 279&47** (follicular fluid)
	(s)ICAM-1	↑↓	↑↓	↑	↓*				B: Up [35] *n* = 20&48; **Up **[36]***n* = 48&49; Down **[30]***n* = 121&232; Down **[37]***n* = 86&170**P: Up [38] *n *= 6&12; Up in peritoneal tissue in menstrual phase, Down in luteal phase [24] *n* = 11&24 E: [26] *n* = 5&5 – O: [27] *n* = 9&9 (cumulus cells)
	IFNɣ	↑↓	↑		↓		↑		B: Up [39] ***n* = 68&70**; Up [13] *n* = 25&19; Down [30] ***n* = 121&232** P: [40] ***n* = 30&50**; [17] *n* = 20&57 – O: [41] *n* = 29&20 (follicular fluid) M: [42] (in the supernatant of menstrual blood derived stem cells with an allogeneic stimulation) *n* = 6&6
	IL6	↑	↑	↑			↑*		B: [43] ***n* = 72&38**; [39] ***n* = 68&70**; [44] ***n* = 31&38**; [12] ***n* = 93&201**; [32] *n* = 17&33; [13] *n* = 25&19; [35] *n* = 20&48; [45] *n* = 22&47; [46] ***n* = 35&45**; [47] ***n* = 60&80**; [48] ***n* = 32&40**; [49] ***n* = 35&43**; [29] ***n* = 103&190**; [14] *n* = 12&75; P: [24] *n *= 11&24; [31] *n* = 22&30; [16] *n* = 16&67; [32] *n* = 17&33; [50] *n* = 28&70; [51] ***n* = 42&36**; [19] = **40&58**; [35] *n* = 20&48; [45] *n* = 22&47 [23]; ***n* = 35&45**; [46] ***n* = 35&45**; [22] ***n *= 45&126**; [48] ***n* = 32&40**; E: [26] *n* = 5&5 – M: [28] *n* = 3&3
	CCL5 (RANTES)	↑	↑	↑					B: [13] *n* = 25&19 – P: [17] *n* = 20&57; [52] *n* = 20&74 – E: [53] *n* = 5&15
	CXCL10 (IP-10)	↓	↑↓	↑*					B: [54] ***n* = 70&77** – E: [55] *n* = 8&8 P: [56] Up ***n* = 32&101**; [33] Up ***n* = 59&73**; Up [38] *n* = 6&12; [54] Down ***n* = 70&77**; [52] *n* = 20&74
Immunity related markers / Cytokines	IL6R	↓*	↑	↓*					B and P: [45] *n* = 22&47 – E: [55] *n* = 8&8
	IL4	↑	↑		↑				B: [13] *n* = 25&19; [47] ***n* = 60&80** P: [57] ***n *= 31&38**; [17] *n* = 20&57 – O: [41] *n* = 29&20 (follicular fluid)
	IL17A	↓	↑↓		↓				B: [58] *n* = 16&27 – P: [52] Up *n* = 20&74 [22], Down ***n* = 45&126** O: [41] *n* = 29&20 (follicular fluid)
	IL2	↑↓	↑		↓				B: Up [13] *n* = 25&19, Down [44] ***n* = 31&38**, Down [58] *n* = 16&27; P: [58] *n* = 15&27 – O: [59] *n* = 5&5 (follicular fluid)
	IL13	↑↓	↓		↑				B: Up [13] *n* = 25&19, Down [60] ***n* = 46&57** P: [21] ***n* = 38&56** – O: [41] *n* = 29&20
	IL10	↑	↑				↑		B: [13] *n* = 25&19; [58] *n* = 16&27; P: [51] ***n* = 42&36**; [23] 2018 ***n* = 35&45** – M: [42] *n* = 6&6
	CCL2 (MCP-1)	↑	↑				↑		B: [61] *n* = 31&18; [62] ***n* = 60&102**; [39] ***n* = 68&70**; [29] ***n* = 103&190**; P: [56] ***n* = 32&101**; [18] *n* = 27&36; [21] ***n* = 38&56** M: [42] *n* = 6&6
	IL9, IL37	↑	↑						B: IL37 [58] *n* = 36&27, IL9: [13] *n* = 25&19, B and P: [48] ***n* = 32&40** P: IL9 [17] *n* = 20&57
	GM-CSF	↑	↑		↓*				B: [13] *n* = 25&19 – P: [17] *n* = 20&57 – O: [27] *n* = 9&9
	IL1β	↑↓	↑				↑*		B: Up [13] *n* = 25&19; [49] Up ***n* = 35&43**, Down [30] ***n* = 121&232** P: [31] *n* = 22&30; [57] ***n* = 31&38**; [17] *n* = 20&57; [33] ***n* = 59&73** M: [28] *n* = 3&3
	IL12	↑↓	↑						B: Up [13] *n* = 25&19, Down [60] ***n* = 46&57** – P: [63] ***n* = 33&72**, [33] ***n* = 59&73**
	FAS/CD95 +	↑↓	↑*						B: [64] Up (soluble) ***n* = 30&30**, [40] Down (T cell surface) ***n* = 30&50**, P: [65] (in NK cells) *n* = 24&46; [66] *n* = 18&26
	IL1α		↑		↑↓*				P: [52] *n* = 20&74 O: [41] Up *n* = 29&20 (folicular fluid), [27] Down *n* = 9&9 (cumulus cells)
	IL3		↓		↑				P [33]: ***n* = 59&73** – O: [41] *n* = 29&20 (folicular fluid)
	CCL22 (MDC)		↑		↓				P: [52] *n* = 20&74 – O: [41] *n* = 29&20 (folicular fluid)
	IL32	↑		↑*					B: [67] ***n* = 35&50** – E: [55] *n* = 8&8
	CD8 + (cytotoxic T cells)	↓		↑					B: [40] ***n* = 30&50**, [68] *n* = 20&54 – E: [69] *n* = 15&15
	CD3 + or CD4 + (T cells)	↑	↑ CD3	↑ CD4					B: [40] ***n* = 30&50**, [68] *n* = 20&54 P: [16] = 16&67 E: [69] *n* = 15&15
	CD25HIGH/FOXP3 + /CD4 + (Treg cells)	↓	↑						B: [70] *n* = 15&17 P: [70] *n* = 15&17, [50] *n* = 28&70; [71] *n* = 25&25
Immunity related markers / Cytokines	CD68 (macrophages)		↑	↑					P [15]: ***n* = 30&48**; [31] *n* = 22&30; [72] *n* = 18&38; [45] *n* = 22&47 E: [73] ***n* = 36&37**
	GAL-3		↑	↓					P: [74] *n* = 8&15 – E: [75] ***n* = 34&34**
	PTGS2			↑	↓				E: [76] *n* = 21&26, O: [77] ***n* = 40&38** (Cumulus cells)
	CXCL12 (SDF-1)	↑			↑*				B: [78] *n* = 10&11, O: [27] *n* = 9&9
ECM/Cell Markers/ Cell fate	**MMP-9 (or MMP-9/NGAL)**	↑	↑		↑	↑	↑*		B: [79] ***n* = 31&60**; [80] *n* = 26&50; [81] ***n* = 140&200** – P [80]: *n* = 26&50 O: [81] ***n* = 140&200**; U: [82] ***n* = 58&73** – M: [28] *n* = 3&3
	MMP-2				↑	↓	↑*		O: [81] ***n* = 140&200** – U: [83] *n* = 25&25; M: [28] *n* = 3&3
	MMP-3	↑	↑	↑					B: [84] *n* = 20&40; [85] *n* = 20&40 P: [24] *n* = 11&24; E: [24] *n* = 11&24, [86] *n* = 20&23
	MMP-7		↑	↑					P: [20] ***n* = 34&124**; E: [87] ***n* = 110&109**
	OSTEOPONTIN, PERIOSTIN	↑	↑	↑					Osteopontin: B and E: [88] ***n* = 41&40 –** P: [38] *n* = 6&12 Periostin: B and P: [89] ***n* = 80&104** – E: [90] *n* = 11&14
	CA-125 aka MUC-16	↑	↑						B: [25] ***n* = 32&71**; [91] ***n* = 37&47**; [92] *n* = 17&35; [93] ***n* = 52&52** P: [94] ***n* = 43&65**; [92] *n* = 17&35; [93] ***n* = 52&52**; [25] ***n* = 32&71** [91]**;** ***n* = 37&47**
	CA-19–9	↑	↑						B: [95] ***n* = 36&50**; [96] ***n* = 40&60**; [91] ***n* = 37&47** – P: [20] ***n* = 34&124**
	CYTOKERATIN-19	↑				↑			B: [97] ***n* = 35&44** – U: [97] ***n* = 35&44**; [98] *n* = 6&11
	**TIMP-1**	↓	↑		↓				B: [81] ***n* = 140&200** – P: [22] ***n* = 45&126** – O: [81] ***n* = 140&200**
	FIBRONECTIN			↓	↑				E: [99] *n* = 18&40 – O: [100] *n* = 10&20
	TGF-β	↑	↑	↑	↑				B: [81] ***n* = 140&200** – P: [50] *n* = 28&70 E: [101] *n* = 10&10 – O: [81] ***n* = 140&200**
	BCL2			↑			↑*		E: [102] *n* = 20&20 – M: [28] *n* = 3&3
	SOX2			↑			↑*		E: [103] *n* = 16&26 – M: [28] *n* = 3&3
Metabolites	GLUTAMINE, ALANINE	↑		↓*	↓				B: Glutamine: [104] *n* = 15&22; Alanine: [105] *n* = 23&22 E: [106] *n* = 24&95 – O: [107] *n* = 9&7 (samples *n* = 50&29)
	LYSINE	↑		↑↓		↓			B: [105] *n* = 23&22 – U: [108] ***n* = 36&45** E: Up [109] *n* = 29&37; Down [106] *n* = 24&95
	LEUCINE	↑		↑↓					B: [105] *n* = 23&22 E Down: [106] *n* = 24&95; Up: [109] *n* = 29&37
	ISOLEUCINE	↓			↑				B: [105] *n* = 23&22 – O: [107] *n* = 9&7 (samples *n* = 50&29)
	THREONINE	↑			↓				B: [105] *n* = 23&22 – O: [107] *n* = 9&7 (samples *n* = 50&29)
	VALINE	↑				↑			B: [105] *n* = 23&22; [110] *n* = 23&50 U: [108] ***n* = 36&45**
Metabolites	TYROSINE			↑	↓				E: [109] *n* = 29&37; [106] *n* = 24&95 O: [107] *n* = 9&7 (samples *n* = 50&29)
	TAURINE			↑		↑			E: [106] *n* = 24&95 – U: [108] ***n* = 36&45**
	LACTATE, 3-HYDROXYBUTYRATE	↑			↑				B: [105] *n* = 23&22 O: [107] *n* = 9&7 (samples *n* = 50&29)
	GLUCOSE	↓		↑*	↓				B: [29] ***n* = 103&190**; [105] *n* = 23&22 – E [106]: *n* = 24&95 O: [107] *n* = 9&7 (samples *n* = 50&29)
Hormones/ Growth Factors	ERα		↑	↑↓			↑*		P: [31] *n* = 22&30 – M: [28] *n* = 3&3 E: [111] Up *n* = 18&38, [112] Down *n* = 26&28
	ERβ		↑	↓					P: [31] = 22&30, E [112]: *n* = 26&28
	LEPTIN	↑	↓*						B: [113] ***n* = 30&30** P: [19] ***n* = 40&58**
	AMH	↓		↑*					B: [114] ***n* = 93&57**, [115] *n* = 17&17 – E: [116] *n* = 20&23
	FGF-2	↑*	↑						P: [52] *n* = 20&74, [117] *n* = 18&38
	VEGF/VEGF-A	↑↓	↑	↑*			↑*		B: Up [30] ***n* = 121&232;** Up [29] ***n* = 103&190;** Up [118] ***n* = 822&1109;** Up [119] ***n* = 30&30;** Down [25] ***n* = 32&71;** Down [60] ***n* = 46&57;** P [22]: ***n* = 45&126** – E: [26] *n* = 5&5 – M: [28] *n* = 3&3
	PDGF-A		↑*	↓					P: [52] *n* = 20&74 – E: [25] *n* = 8&15
	ACTIVI*N*-A	↑*		↑*					B: [120] ***n* = 75&139** – P: [121] *n* = 3&6
miRNAs	**MIR-451/MIR-451A**	↑↓	↑	↑	↓				B: Up [122] n = 24&24; Up [123] **n = 99&89**; Down [124] **n = 66&80** P: [22] **n = 45&126** – E: [125] n = 20&40 – O: [126] **n = 30&30** (follicular fluid)
	MIR-122, MIR-199A	↑	↑						B: [127] *n* = 25&60, [46] ***n* = 35&45** – P: [46] ***n* = 35&45**
	MIR-15A-5P	↓		↓					B: [128] ***n* = 30&60** – E: *n* = 3&3
	MIR-17/MIR-17-5P	↓		↓					B: [47] ***n* = 60&80**; [128] ***n* = 30&60** – E: [129] ***n* = 51&51**
	MIR-135A	↑↓						↑	B: Down [130] *n* = 24&24; Up [131] *n* = 17&17 – Sa: [131] *n* = 17&17
Other	GLYCODELIN-A	↑	↑						B: [37] ***n* = 86&170**; [30] ***n* = 121&232** [19]**;** ***n* = 42&57**; [35] *n* = 20&48 P: [37] ***n* = 86&170**; [19] ***n* = 42&57**; [32] *n* = 17&33, [35] *n* = 20&48
	HAPTOGLOBIN	↓	↑						B: [132] *n* = 15&15 – P: [133] *n* = 8&16
	PGP9.5		↑	↑*					P: [72] *n* = 18&38 – E: [134] *n* = 20&20

Arrows indicate the direction of change in endometriosis patients. *: change detected only in a subgroup of patients (either because of patient inclusion criteria restricting to a subgroup of patients, or detected only in a specific subgroup of all included patients). *ILX* Interleukin X, *CA-X* Cancer Antigen X, *MMP-X* Matrix Metalloproteinase-X, *CDX* Cluster of Differentiation X, *CXCLX* Chemokine (C-X-C motif) ligand X, *TNFα* Tumor Necrosis Factor alpha, *(s)ICAM-1* (soluble) Intercellular adhesion molecule-1, *IFNy* Interferon gamma, *CCL5* Chemokine (C-C motif) ligand 5, *IP-10* Interferon gamma induced protein 10, *IL6R* Interleukin 6 receptor, *MCP-1* Monocyte Chemoattractant protein-1, *GM-CSF* Granulocyte/macrophage-colony stimulating factor, *MDC* Macrophage-derived chemokine, *GAL-3* Galectin-3, *VEGF* Vascular Endothelial Growth Factor, *NGAL* Neutrophil Gelatinase-Associated Lipocalin, *TIMP-1* TIMP metallopeptidase inhibitor-1, *MUC-16* Mucin-16, *TGF-β* Transforming growth factor beta, *BCL2* B-cell lymphoma 2, *ERα* Estrogen receptor alpha, *ERβ* Estrogen receptor beta, *AMH* Anti-Müllerian hormone, *FGF-2* Fibroblast growth factor 2, *PDGF* Patelet-derived growth factor, *PGP9.5* Protein gene product 9.5, *PTGS2* Prostaglandin-endoperoxide synthase 2, *SDF-1* Stromal cell-derived facotr-1.

### Cohort sizes are relatively small except for peripheral blood

**Fig. 3:**
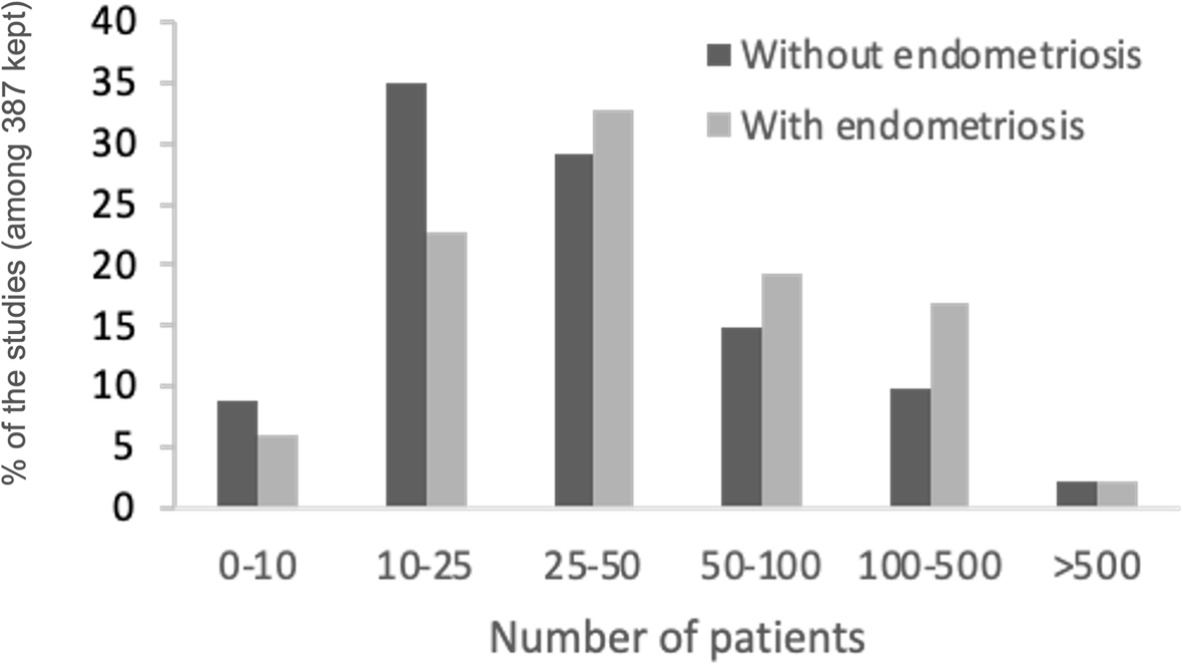
Cohort size distribution. Analyses performed among the 387 articles kept and illustrating the distribution of studies according to cohort size

The majority of selected studies included between 10 and 50 women in each group with and without endometriosis (Fig. 3). Unsurprisingly, all studies including more than 500 women per group focused on peripheral blood. Biological compartments are also very unequally studied in cohorts with 100 to 500 women per group: 84.3% studied peripheral blood, 8.6% eutopic endometrium, 5.7% peritoneal fluid and 1.4% follicular fluid. Biological compartments that can be assessed non-invasively (urine, menstrual blood, saliva, feces, cervical mucus), have not been studied on a large scale.

### Only 4 reproducible biomarkers have been consistently detected across tissues in large cohorts

We highlighted 4 candidate biomarkers identified by at least two different research teams in 3 biological compartments or more, with at least one well-powered study per compartment including 30 or more controls and 30 or more patients with endometriosis (Table 2). Here, we summarized the main lines of evidence supporting each of these 4 biomarkers as potential diagnostic elements for endometriosis.

**Table 2:** Candidate biomarkers found by independent teams in at least 3 biological compartments in large cohort

Biomarker	Patients		Potential confunding factors		Type of measured molecule related to the indicated biomarker	Main results		Missing information	Adjustements				Statistics as regards diagnostic accuracy			References
	Control group (n)	Endometriosis group (n)	Age	BMI (endometriosis patients vs controls)		Biological sample type	Global variation (endometriosis patients vs control)	Concerning endometriosis phenotype, cycle phase, hormonal treatments or symptoms	Endometriosis subtype	Cycle phase	Symptoms	Hormonal treatments	AUC	Sensitivity	Specificity	
TNF-alpha	Healthy volunteers without detected endometriosis by ultrasound examination (*n* = 103)	Surgically and histologically proven ovarian endometriosis (*n* = 190) Stage I/II (*n* = 77) and III/IV (*n* = 113)	NS	ND	Protein	Serum	Increased 1.4 times	Cycle phase, treatment for controls, symptoms	Higher in stage III/IV vs I/II	NA	ND or NA	ND or NA	0.776 (alone) 0.913 (with VEGF, sFlt-1, IL6 and MCP1)	ND (alone) 85.79% (with VEGF, sFlt-1, IL6 and MCP1)	ND (alone) 87.38% (with VEGF, sFlt-1, IL6 and MCP1)	[29]
	Without endometriosis (laparoscopic exam) (*n* = 93)	Endometriosis patients (*n* = 201) Stage I/II (*n* = 132) and III/IV (*n* = 69)	ND	ND or NA	Protein	Plasma	Reduced 14.6 times	Pain symptoms, lesions localisations	Significant reduction for I/II and III/IV vs controls NS I/II vs III/IV	Change detected in all and secretory phase	ND or NA	NP	0.758 (all phase) 0.787 (secretory)	79.5% 80.6%	73.7% 73.7%	[12]
	Without endometriosis (laparoscopic exam) (*n* = 121)	Endometriosis patients (*n* = 232) Training subset (*n* = 155), test subset (*n* = 67) Stage I/II (*n* = 148) and III/IV (*n* = 84) US negative (*n* = 175)	NS	ND or NA	Protein	Plasma	Reduced 1.2 times (only in training set)	Lesions localisations	ND (for stages)	Change detected in all and proliferative phase	ND	NP	0.65 (for training set US negative patients in follicular phase)	78%	57%	[30]
	Without endometriosis (laparoscopic exam) (*n* = 35)	Surgically and histologically proven endometriosis (*n* = 45) Stage I/II/III/IV (*n* = 10/8/18/9) PE (*n* = 39), OE (*n* = 18), DIE (*n* = 18)	NS	Lower	Protein	Plasma	NS	Pain symptoms other than dysmenorrhea	Higher in patients with vs without DIE	NS (between phase)	NS	ND	ND	ND	ND	[23]
						Peritoneal fluid	Increased ~ 2 times									
	Without endometriosis (laparoscopic exam) (*n* = 59)	Endometriosis (*n* = 73) Stage I/II (*n* = 31) a*n*d III/IV (*n* = 42) PE (*n* = 17), OE (*n* = 30), DIE (*n* = 14)	NS	NS or NA	Protein	Peritoneal fluid	NS (All endometriosis) Increased 3.39 times (OE, proliferative phase only)	None	Higher in OE vs DIE (all phases) Higher OE vs PE (proliferative phase)	Main result only found in proliferative phase	ND	NP	ND	ND	ND	[33]
	Infertile patients undergoing ICSI without endometriosis as assessed by laparoscopy (*n *= 279)	Surgically and histologically proven endometriosis, infertile patients undergoing ICSI (*n* = 47) Stage I/II (*n* = 33) and III/IV (*n* = 14)	NS	NS	Protein	Follicular fluid	Increased 2 times	Pain symptoms, lesions localisations	ND	NP	NA	NP	ND	ND	ND	[34]

Biomarker	Patients		Potential confunding factors		Type of measured molecule related to the indicated biomarker	Main results		Missing information	Adjustements				Statistics as regards diagnostic accuracy			References
	Control group (n)	Endometriosis group (n)	Age	BMI (endometriosis patients vs controls)		Biological sample type	Global variation (endometriosis patients vs control)	Concerning endometriosis phenotype, cycle phase, hormonal treatments or symptoms	Endometriosis subtype	Cycle phase	Symptoms	Hormonal treatments	AUC	Sensitivity	Specificity	
MMP-9 or MMP-9/NGAL	Healthy volunteers without endometriosis (laparoscopic exam) (*n* = 31)	Surgically proven endometriosis (*n* = 60) OE (*n* = 31)	NS	NS	Protein	Serum	Increased 1.25 times	Cycle phase and symptoms	Higher in stage III/IV vs I/II	NA	NA	NP	Predictive accuracy of MMP-9 for severe endometriosis (threshold: 14.13 pg/ml) 0.878	80%	73.3%	[79]
	Infertile women without endometriosis but with tubal-factor infertility undergoing IVF (*n* = 200)	Infertile women with endometriosis confirmed by laparoscopy and histological analysis, undergoing IVF Stage III/IV (*n* = 140)	NS	NS	Protein	Serum	Increased 1.5 times	Pain symptoms, lesions localisations	NP	NP	NA/NP for infertility	Progesterone supplementation decrease (1.4 times) MMP-9 serum level in pregnant women	ND	ND	ND	[81]
						Follicular fluid	Increased 1.5 times									
	Surgically and histologically or clinically verified no evidence of endometriosis (*n* = 58)	Surgically and histologically verified endometriosis or clinical diagnosis of endometriosis (*n* = 73)	NA	NA	Protein	Urine	NA	Cycle phase, hormonal treatments, symptoms and lesions localisations	NP	NA	NA	NA	The odds ratio of having MMP-9 or MMP-9/NGAL in the urine and having endometriosis was 7.8 (95% CI: 2.5–25.1; p < 0.001) and 6.3 (95% CI: 1.7–22.8; p < 0.001), respectively	ND	ND	[82]
TIMP-1	Infertile women without endometriosis but with tubal-factor infertility undergoing IVF (*n* = 200)	Infertile women with endometriosis confirmed by laparoscopy and histological analysis, undergoing IVF Stage III/IV (*n* = 140)	NS	NS	Protein	Serum	Reduced 1.2 times	Pain symptoms, lesions localisations	NP	NP	NA/NP for infertility	NP	ND	ND	ND	[81]
						Follicular fluid	Reduced 1.2 times									
	Healthy volunteers without endometriosis (laparoscopic exam) (*n* = 45)	Surgically proven endometriosis (*n* = 126)	NA	NA	Protein	Peritoneal fluid	Increased 1.9 times	Pain symptoms, lesions localisations	No statistical difference between stage I/II and III/IV	Increase only in secretory phase	NA/Same range of increase regardless of fertility status	NP	ND	ND	ND	[22]

Biomarker	Patients		Potential confunding factors		Type of measured molecule related to the indicated biomarker	Main results		Missing information	Adjustements				Statistics as regards diagnostic accuracy			References
	Control group (n)	Endometriosis group (n)	Age	BMI (endometriosis patients vs controls)		Biological sample type	Global variation (endometriosis patients vs control)	Concerning endometriosis phenotype, cycle phase, hormonal treatments or symptoms	Endometriosis subtype	Cycle phase	Symptoms	Hormonal treatments	AUC	Sensitivity	Specificity	
miR-451/miR-451a	Healthy volunteers without endometriosis (laparoscopic exam) (*n* = 99)	Surgically proven endometriosis (*n* = 89) Stage I/II/III/IV (*n* = 27/17/36/19)	NS	NS	miRNA	Serum	Increased 4.5 times (qRT-PCR)	Symptoms, lesions localisations	Significant increase for I/II and III/IV vs controls NS I/II vs III/IV	NS	NA	NS	0.84 (alone)	90%	72.9%	[123]
													0.939 (in combination with miR-125b, miR-150, miR-342, miR-3613 and let-7b)	83%	96%	
	Healthy volunteers without endometriosis (*n* = 66)	Infertile women with endometriosis confirmed by laparoscopy and histological analysis, undergoing IVF/ICSI (*n* = 80) Stage I/II (60) and III/IV (20)	NS	NS	miRNA	Serum	Decreased 1.7 times (qRT-PCR)	Other symptoms than infertility for patients, symptoms and treatment for control, cycle phase, lesions localisations	NP	NA	NA	NA	0,978	ND	ND	[124]
	Without endometriosis (laparoscopic exam) (*n* = 45)	Surgically proven endometriosis (*n *= 126)	NA	NA	miRNA	Peritoneal fluid	Increased 2.5 times (qRT-PCR)	Pain symptoms, lesions localisations	No statistical difference between stage I/II and III/IV	Significantly up-regulated during menstrual phase compared to proliferative and secretory phase in endometriosis women but not in control	NA/No impact of the fertility status	NP	ND	ND	ND	[22]
	Infertile women without endometriosis (laparoscopic exam), undergoing IVF (*n* = 30)	Infertile women with endometriosis confirmed by laparoscopy, undergoing IVF Stage III/IV All with OE (*n* = 30)	NS	NS	miRNA	Follicular fluid	Decreased 2 times (qRT-PCR)	Pain symptoms	NP	NP	NA/NP for infertility	NP	ND	ND	ND	[126]

*ND*: Not disclosed/done (despite available information), *NA* Not available, *NS* No significant difference/correlation, *NP*: Not possible (same for all samples), *PE* Peritoneal Endometriosis, *OE* Ovarian Endometriosis, *DIE* Deep Infiltrating Endometriosis, *ICSI* IntraCytoplasmic Sperm Injection, *IVF* In Vitro Fertilization, *US* Ultrasound, *VEGF* Vascular Endothelial Growth Factor, *sFLt-1* Soluble Fms-like tyrosine kinase 1, *MCP1* Monocyte Chemoattractant protein 1, *Il6* Interleukin 6, *TNF-alpha* Tumor Necrosis Factor-alpha, *MMP-9* Matrix Metalloproteinase-9, *NGAL* Neutrophil Gelatinase-Associated Lipocalin, *TIMP-1* TIMP metallopeptidase inhibitor 1

### TNF-α

Tumor necrosis factor alpha (TNF-α), a pro-inflammatory cytokine, was consistently reported as increased in larger cohorts of women with endometriosis in two biological compartments: peritoneal fluid [23, 135], and follicular fluid [34]. In the peritoneal fluid, this increase was further consolidated by consistent results from smaller cohorts [17, 31, 32], although two other studies found no significant differences between women with and without endometriosis [21, 51]. How TNF-α changes in peritoneal fluid tie in with endometriosis phenotypes and menstrual phases was unclear, with reported increases in both stages I/II and III/IV [32], only in stage III/IV with no difference between proliferative and secretory phases, or only in patients with endometrioma and in proliferative phase [135] – some of which may reflect inappropriate statistical power to control for Type II errors when stratifying cohorts. Another increase was also detected in the endometrium at the mRNA level during menstrual phase [24]. While TNF-α was also reported as modified in blood, results were inconsistent, reporting increases [13, 29], decreases [12, 30] or no change [32, 39, 44, 60, 67], both within small or large cohorts. When focusing on large cohorts, increases were observed in serum while the decreases were in plasma, suggesting an importance on the blood collection method (Table 2). Unfortunately, diagnostic accuracy of TNFα was only assessed in blood and yielded low specificity and sensitivity, which is unsurprising in light of the discrepancies across studies.

### MMP-9 or MMP-9/NGAL

Enzyme matrix metalloproteinase (MMPs), including MMP-9, are involved in extracellular matrix remodeling via proteolytic activity. They play a key role in physiological (like embryogenesis and wound healing) and pathophysiological (invasion and tissue destruction mechanisms) uterine processes [80]. In this review, we observed that MMP-9 levels appeared to be increased in endometriosis in all studies and regardless of the biological compartment studied. Interestingly, fertility status and menstrual cycle phases do not seem to affect the variations of this biomarker [79–82, 136]. Although still to be confirmed, the diagnostic value of this biomarker seems to be appropriate [79, 82]. We note that a therapeutic approach to reduce MMP-9 level through progesterone supplementation to improve IVF success rates in endometriosis patients showed promising success, suggesting that MMP-9 may have treatment as well as diagnosis value in endometriosis [81].

### TIMP-1

TIMP-1, a metalloproteinase inhibitor, is involved in extracellular matrix remodeling which is particularly intense in ovary during follicular development and cyst formation and in endometrium during dynamic cyclic changes across the menstrual cycle [81]. TIMP-1 showed inconsistent regulation between different biological compartments in women with endometriosis, with reported decreases in blood and ovarian tissue and an increase in peritoneal fluid across well-powered cohorts [22, 81]. Although this remains to be confirmed, this candidate biomarker does not appear discriminative for disease stage and fertility status, but seems impacted by menstrual cycle phases [22].

### miR451/miR451a

MiRNAs are small endogenous noncoding functional RNAs [122]. As they are released into the circulation, their interest as biomarkers has been the subject of numerous studies, specific miRNA expression patterns are hallmarks for numerous diseases [122, 123]. These associations between miRNA expression profiles and diseases are often obtained by non-targeted screening (microarray or miRNome sequencing), and their mechanistic roles in physiology or pathophysiology are poorly studied. miR-451 seemed to perform well as a diagnostic marker of endometriosis across different biological fluids and study settings, particularly in combination with other miRNAs [123, 124]. This biomarker does not seem to be correlated with endometriosis severity [22, 123]. Understanding the observed discrepancies in the direction of variation will require studies with systematic adjustments for disease severity, menstrual cycle phases, treatments, symptoms, clinical characteristics of the cohorts and associated comorbidities.

## Discussion

Endometriosis biomarker research investigated a large variety of biological compartments so far, some of which are relevant to the local mechanisms of endometriosis pathophysiology (eutopic endometrium, peritoneal fluid, ovary, menstrual blood, cervical mucus), while others approached endometriosis as a systemic disorder resulting in body-wide dysregulations (peripheral blood, urine, feces, saliva). A first valuable outcome of our review was that non-invasively accessible biological compartments (urine, menstrual blood, feces, saliva, cervical mucus) remained drastically understudied despite their potential to transform endometriosis diagnosis. These compartments can address disease modifications both at the systematic and local levels, and deserve more focused attention in the future.

We identified a total of 1107 candidate biomarkers across all nine studied biological compartments, suggesting that endometriosis is potentially associated with widespread molecular modifications. However, agreement between studies, protocols and laboratories was strikingly low, with few candidate markers consistently modified within the same compartment and exhibiting similar directions of change. This suggests that many reported candidate biomarkers were either highly dependent on technical considerations, or represent false positives due to unaccounted confounders. Only 4 of these candidates were reproducibly detected across several compartments by different research teams and with appropriately powered cohorts, and we argued that these markers with widespread modifications should be first-line candidates for investigation in more accessible biological compartments.

As highlighted in this review, the relationships between marker variations and biological compartments were often obscured by uneven consideration of fundamental variables such as disease stage, symptoms, treatments, and menstrual cycle phase. Symptoms and treatments were the major missing elements in many study designs. These variables were rarely analyzed and often absent altogether. Regarding symptomatic treatments (painkillers, anti-inflammatory drugs), neither their effectiveness nor their frequency of use was reported. Menstrual cycle phases were also key variables as many metabolic and regulatory pathways vary throughout the cycle, including one-carbon metabolism [137] and miRNAs [128, 138], but were frequently overlooked. Most articles used the revised ASRM classification to rank phenotypes from endometriosis stage I (minimal) to stage IV (severe) [2]. Deep infiltrating endometriosis is then classified as stage III or IV regardless of the presence of endometrioma. However, presence of endometriomas seems decisive for some biomarkers regulation, especially metabolites. A more accurate classification like ERZIAN scoring may allow for better discrimination between different disease phenotypes [2]. All these parameters may contribute to explain the lack of reproducibility between studies, and standardizing data records may help alleviate this issue in future studies.

The top 4 candidate biomarkers of interest identified here belong to different molecular categories (miRNAs, extra-cellular matrix, and cytokines), and are involved in pathophysiological processes common to many diseases, especially extra-cellular matrix remodeling and inflammation. Previous studies have generally combined elements of the same molecular category together [122, 123], but combinations involving different molecular families are more rarely studied [30]. The 4 candidate biomarkers identified in this work were present in blood, an accessible and relevant biological compartment for diagnostic test development. They have never been combined together to test their diagnostic performance in endometriosis, but their association should be evaluated. Comparing sensitivity and specificity across studies to identify potential combinations of markers of interest remains difficult, as designs and cut-offs varied between studies and between biological compartments. We noted that formal meta-analyses of endometriosis biomarkers were largely absent, and will likely remain challenging due to the heterogeneity in study designs and data collection records that we highlighted above, limiting the reusability of available information.

Finally, this systematic study also came with some limitations. First, and despite our best efforts, we may have missed biomarkers that meet our selection criteria but are listed under different aliases during manual literature curation. We however expect that these instances were rare and did not affect the overarching conclusions of this study. Another important limiting factor was the design of the selected studies, which typically excluded rather than accounted for stratifying parameters of interest. Most of the highlighted adjustments were by exclusion of other categories of patients, for example by including only a single endometriosis phenotype in the cohort, or enrolling women in the same phase of the cycle. In this context, rigorously assessing the impact of adjustment and the differential effects of endometriosis subtypes, cycle phases, symptoms and treatments on biomarker levels remained challenging. Another potential source of bias was the heterogeneity of the control groups, a problem widely recognized for endometriosis research. Indeed, supposedly healthy donors may contain asymptomatic endometriosis patients, while most laparoscopically examined controls with confirmed absence of endometriosis had other gynecological or fertility issues. In most cases, these women presented benign comorbidities (e.g. leiomyomas, ovarian cysts) which were not matched with the case group and may impact the levels of certain markers. While these markers were also of interest to eliminate other diseases during endometriosis diagnosis, they addressed a separate question compared to diagnosing endometriosis at large in the population. A final issue that may interfere with reproducibility concerned the methodology of the studies. At this time, few untargeted studies with large discovery and validation cohorts used omics technologies for high-throughput biomarker discovery. The majority of studies focused on a limited panel of predefined targets and many potential biomarkers were therefore not evaluated. We chose to focus on biomarkers reported by independent research teams and in multiple tissues to improve the relevance and the strength of evidence, but numerous biomarkers were probably unconfirmed because they have not been evaluated so far by independent team and in several compartments.

## Conclusion

It appears necessary to rethink endometriosis candidate biomarkers research by designing studies that can be integrated at different levels: i) local and systemic biological compartments; ii) different disease phenotypes with improved characterisation; iii) treatments and their impacts; iv) symptoms; and v) menstrual cycle phases. Access to these parameters will require harmonisation of data collection methods following recommendations of the EPHect project [139]. Such harmonisation would enable meta-analyses, yield a considerable increase in cohort sizes, and facilitate investigations into the effects of these stratifying variables. As endometriosis biomarker discovery remains challenging, sensitivity may be improved by combining biomarkers from different molecular pathways. However, combining biomarkers across biological compartments seems unsustainable in clinical practice, and identifying the most relevant biological compartment remains an important challenge. To this regard, our study pinpoints numerous discrepancies in the results obtained in peripheral blood. Local approaches may lead to more consistent results, as is the case in peritoneal fluid, which can unfortunately not be assessed non-invasively. We therefore highlight the need to further investigate non-invasively accessible biological fluids, especially locally accessible such as menstrual fluid or cervical mucus.

## Supplementary Information

**Additional file 1: Additional material. **PRISMA checklist

**Additional file 2: Additional Table 1. **References of the 447 selected articles

**Additional file 3: Additional Table 2. **Quantitative summary of significantly modified biomarkers in endometriosis identified by biological compartment

## Data Availability

All data generated or analysed during this study are included in this published article and its supplementary information files. The code will be made available on request.

## Abbreviations

(r)ASRM: (The revised) American Society for Reproductive Medicine
MeSH: Medical Subject Headings
TNF-a: Tumor Necrosis Factor alpha
MMP-9: Enzyme Matrix MetalloProteinase 9
NGAL: Neutrophil Gelatinase-associated lipocalin
TIMP-1: TIMP metallopeptidase inhibitor
miR: Micro RNA
MRI: Magnetic Resonance Imaging
HGNC: HUGO Gene Nomenclature Committee
